# Ultrastructure of the Spermiogenesis in *Halyomorpha halys* (Hemiptera: Pentatomidae): X-Irradiation and New Insights on the Centriolar Region Organization

**DOI:** 10.3390/insects15070505

**Published:** 2024-07-05

**Authors:** Francesco Paoli, Massimo Cristofaro, Gerardo Roselli, Raffaele Sasso, Sergio Musmeci, Francesco Barbieri, Chiara Sciandra, Valentina Vanoni, Loris Menegotti, Pio Federico Roversi, Gianfranco Anfora, David Mercati, Romano Dallai

**Affiliations:** 1 CREA Research Centre for Plant Protecion and Certification, Via di Lanciola 12/a, 50125 Firenze, Italy; francesco.paoli@crea.gov.it (F.P.); francesco.barbieri@crea.gov.it (F.B.); chiara.sciandra@crea.gov.it (C.S.); 2 Biotechnology and Biological Control Agency (BBCA), Via A. Signorelli 105, 00123 Roma, Italy; m.cristofaro55@gmail.com (M.C.); gerardo.roselli@unitn.it (G.R.); 3 Center Agricolture Food Enviroment, University of Trento, Via Edmund Mach 1, 38098 Trento, Italy; gianfranco.anfora@unitn.it; 4 SSPT-BIOTEC Laboratory, Italian National Agency for New Technologies, C.R. Casaccia, Via Anguillarese 301, 00123 Roma, Italy; raffaele.sasso@enea.it (R.S.); sergio.musmeci@enea.it (S.M.); 5 Department of Life Sciences, University of Siena, Via Aldo Moro 2, 53100 Siena, Italy; david.mercati@unisi.it; 6 Azienda Provinciale per i Servizi Sanitari, 38122 Trento, Italy; valentina.vanoni@apss.tn.it (V.V.); loris.menegotti@apss.tn.it (L.M.); 7 National Reference Institute for Plant Protection, Via Bertero 22, 00156 Roma, Italy; piofederico.roversi@crea.gov.it

**Keywords:** atypical MTOC, centrioles, insect sperm, pest control, SIT, spermatogenesis, sterile insect technique, virus

## Abstract

**Simple Summary:**

The brown marmorated stink bug, *Halyomorpha halys*, native to Asia, is now an insect pest in several countries. The sterile insect technique (SIT), involving the sterilization of insects by ionizing radiation and field release, is one method to control insect pest. This study examines the ultrastructure of *H. halys* spermiogenesis in both irradiated and wild males. Typical hemipteran sperm features were observed, such as mitochondrial derivative–axoneme bridges, the absence of accessory bodies, crystalline inclusions in mitochondrial derivatives, an acrosome with packed tubules, and a plaque-shaped microtubular organizing center (MTOC) in the centriolar region. Occasionally, two centrioles were observed in spermatids, with one disappearing later—a novel feature for insect spermiogenesis. In irradiated adults, cysts exhibited inconsistent radiation effects, including disorganized sperm arrangement, incomplete sperm cell divisions, multiple organelle copies within cells, failure to reabsorb cytoplasm, and a lack of axonemes. Additionally, rod-shaped viruses or virus-like particles were found in vasa deferentia, regardless of irradiation.

**Abstract:**

*Halyomorpha halys* (Heteroptera: Pentatomidae) is an insect pest native to Asia that has spread over the last two decades to most of the North America, parts of South America, Europe and North Africa. Its impact is significant as it can feed on more than 300 host plants, rendering affected fruits and vegetable crops unsellable or of lower quality. Various chemical and biological methods have been used to control this pest, with varying degrees of success. The sterile insect technique (SIT) is a pest control method involving the sterilization of insects via ionizing radiation and their subsequent mass release into the field. In the present contribution, the spermiogenesis of *H. halys* was studied from an ultrastructural point of view in both irradiated and non-irradiated adult males. In both cases, we observed ultrastructural characteristics typical of hemipteran sperm cells: bridges connecting the mitochondrial derivatives and the axonemal microtubules, the absence of accessory bodies, and the presence of two or three crystalline inclusions within the mitochondrial derivatives, an acrosome composed of tightly packed tubules, and an atypical, plaque-shaped microtubular organizing center (MTOC) in the centriolar region. Moreover, in the same region, we seldom observed the presence of two centrioles in the spermatids, one of which disappeared at a later stage of maturation. This feature is a novelty for insect spermiogenesis. The cysts of irradiated adults were not all uniformly affected by the radiation. However, irradiated cysts sometimes exhibited a general disorganization of sperm arrangement, incomplete divisions of sperm cells resulting in multiple copies of the same organelle within the same cell, failure to reabsorb the cytoplasm, and the lack of axonemes. Finally, rod-shaped viruses or virus-like particles were observed in vasa deferentia independently of irradiation.

## 1. Introduction

The brown marmorated stink bug (BMSB) *Halyomorpha halys* (Heteroptera: Pentatomidae) is a pest native to Asia that invaded mid-Atlantic regions since the 1990s, causing severe damages to fruits and some vegetal crops [1,2,3,4,5].

Although several chemical and biological control agents have been used worldwide to limit crop losses, so far, the control of *H. halys* is somewhat unsatisfactory [6]. In this scenario, it is pivotal to test new strategies to curtail the impact of this alien bug pest on agricultural productions.

The sterile insect technique (SIT) is a species-specific pest control method involving the mass rearing, sterilization, and inundative releases of sterile insects (usually males) of the same pest species [7]. This strategy uses irradiation, such as γ-rays and X-rays, to sterilize large numbers of the target pest species rendering, them sexually competitive but unable to produce offspring [8,9]. Wild females that mate with sterile males do not produce offspring, thereby reducing the insect pest population [7,8,9]. Due to its highly host-specific approach, SIT is considered one of the most environmentally friendly insect pest control methods ever developed [8,9].

However, SIT has only been applied to few insect orders: most of the applications were addressed mainly to Lepidoptera and Diptera, while the irradiation biology of Hemiptera remains to be fully investigated. The main reason why field applications of SIT with sterile phytophagous adult hemipterans have not been applied is closely related to the fact that the irradiated bugs could cause unwanted damage to host crop species [10].

Nevertheless, for many hemipteran pest species, irradiation to induce egg sterility can be synergistically combined with classical biological control methods using co-evolved egg parasitoids. Interest in evaluating the feasibility of using irradiation techniques to support classical biological control for the managing of *H. halys* emerged from the concept that these strategies focus on egg parasitoids for pentatomid pests [11,12,13]. Recent data confirm that sterile eggs, produced by mating irradiated males with fertile females, can be used in a classical biological control context to enhance pest management in a synergic manner [14,15,16,17].

To better evaluate the process that makes this technique effective, it is important to know the reproductive biology of the pest and, in particular, how spermatogenesis occurs in wild insects and how it could be affected by irradiation.

The presence of bridges that link the mitochondrial derivatives and the axonemal microtubules, the absence of accessory bodies and the presence of two or three crystalline inclusions within the mitochondrial derivatives characterize the sperm of Heteroptera [18].

Furthermore, the centriolar region of Heteroptera shows the peculiarity of an atypical microtubular organizing center (MTOC), which is quite unusual for that region in insects, and whose formation and function need to be accurately investigated [19,20,21].

In addition, with regard to morphology, within the Pentatomidae family, it has been observed that sperm of different length and size may coexist in the same ejaculate [22,23,24].

In this study, we aim to investigate spermiogenesis in *H. halys*, focusing on the development of structures related to the centriolar region and paying particular attention to assessing the potential effects of irradiation on sperm cell maturation.

## 2. Materials and Methods

### 2.1. Origin and Rearing of the Insect

*Halyomorpha halys* originated from the mass-rearing facilities of ENEA SSPT-BIOTEC C.R. Casaccia, Rome (Italy), from adults collected using traps in May 2018 in Rome, Italy (42°2′36.96″ N, 12°17′53.66″ E). Two other colonies have been established with the same insect population at the laboratories of the Fondazione Edmund Mach (FEM), Trento (Italy), and CREA DC, Florence (Italy), following the same rearing protocol described below. Insects were reared in cloth cages (30 × 30 × 30 cm; BugDorm^®^, Taichung, Taiwan) and fed with hazelnuts, green beans and wet cotton for a water source. The insects were kept in a climatic room (26 ± 1 °C, 70–80% RH, 12:12 L:D) until death.

### 2.2. Light Microscopy

Several male adult specimens were anesthetized and dissected. Dissection took place in a phosphate-buffered solution (0.1 M phosphate buffer pH 7.2) with added sucrose (3%). Then, the reproductive system was isolated and examined under both stereo and light microscopes (Nikon SMZ 1500, Nikon, Tokyo, Japan and Nikon Eclipse TE 2000-U, Nikon, Tokyo, Japan) to understand its overall organization. To reveal the sperm nucleus, samples were prepared for epifluorescence analysis: fresh materials from the testes and/or vasa deferentia were dissected in phosphate-buffered saline (PBS) pH 7.4 and then treated with Hoechst 1% in PBS for 5 min. Finally, the specimens were mounted in glycerol and observed under a Nikon Eclipse TE 2000-U light microscope. All images were taken with a Nikon digital camera.

### 2.3. Immunofluorescence Preparations

Testes were dissected from adults of *Halyomorpha halys* specimens and processed for γ-tubulin and DNA visualization. Dissection was performed in phosphate-buffered saline (PBS) followed by mechanical disruption via squashing under coverslip. To preserve the tissue morphology, samples were rapidly frozen in liquid nitrogen. After removal of the coverslip, the samples were subjected to a 10 min methanol immersion at −20 °C for permeabilization. The γ-tubulin localization was achieved through a series of incubation steps: washing in PBS for 15 min to remove residual methanol; incubation in PBS containing 0.1% bovine serum albumin (PBS-BSA) for 1 h at room temperature to block non-specific antibody binding; overnight incubation at 4 °C with a 1:100 dilution of mouse anti-γ-tubulin-GTU88 primary antibody (Sigma Aldrich, St. Louis, MO, USA) in a humidified chamber; washing with PBS-BSA to remove unbound primary antibody; and incubation with Alexa Fluor 488 (Invitrogen) secondary antibody diluted 1:800 in PBS-BSA for 1 h at room temperature for specific labeling of γ-tubulin. DNA was visualized by a subsequent 3–4 min Hoechst stain incubation. Finally, the processed testes were mounted on slides using a 90% glycerol solution in PBS and imaged using a Carl Zeiss Axio Imager Z1 microscope (Carl Zeiss, Oberkochen, Germany) equipped with a Carl Zeiss AxioCam HR cooled charge-coupled device (CCD) camera (Carl Zeiss, Oberkochen, Germany). The initially acquired grayscale images were subsequently pseudocolored and merged using Adobe Photoshop CS3 software (Adobe Systems).

### 2.4. Transmission Electron Microscopy (TEM)

The male genital system underwent dissection and overnight fixation in 2.5% glutaraldehyde prepared in phosphate buffer (PB). Following thorough rinsing with PB, the tissue was post-fixed in 1% osmium tetroxide (OsO4) for a two-hour duration. Dehydration of the genital system was achieved through a graded ethanol series, followed by embedding in a mixture of Epon-Araldite resins. A subset of semi-thin sections obtained using a PowerTome ultramicrotome (RMC, Tucson, Arizona, USA) were stained with 1% toluidine blue for 1 min and subsequently examined under a Nikon Eclipse TE 2000-U light microscope (Nikon, Tokyo, Japan). The remaining semi-thin sections were directly observed under the same light microscope. Routine staining of thin sections involved uranyl acetate and lead citrate, followed by examination using Philips CM 10 (Philips, Amsterdam, The Netherlands) and Jeol Jem 1011 electron microscopes (Jeol Ltd., Tokyo, Japan) operating at an accelerating voltage of 80 kV. Images were captured using an Olympus Veleta (Olympus, Hamburg, Germany) digital camera.

### 2.5. X-Irradiation

On the day of emergence, groups of ten male insects were confined in two 9 cm diameter Petri dishes. The Petri dishes containing the insects arranged for irradiation were transported from rearing colonies of FEM to the laboratories of the Radiation Oncology Department at Santa Chiara Hospital in Trento, Italy. Following the irradiation protocol described in Roselli et al. (2023) [17], the insects were X-irradiated using an Elekta Precise Sli linear accelerator (LINAC) from Elekta AB (Stockholm, Sweden), with high-energy photons (energy used: 6 MV); the dose rate applied was 250 cGy/min and the insects absorbed a radiation dose of 40 Gy. After irradiation, the insects were transported using cloth cages (24.5 × 24.5 × 24.5 cm; BugDorm^®^, Taichung, Taiwan) to the CREA laboratories in Florence (Italy) for the analysis on the ultrastructure of the spermiogenesis.

The insects from the control treatment group (0 Gy) were provided by ENEA and transported to CREA for the same study on the spermiogenesis. The insects were exposed to the same environmental conditions as the irradiated group (see Section 2.1) to exclude any effects on the survival rate due to the different rearing conditions of the insects.

### 2.6. Statistical Analysis

The lengths of the sperm of *Halyomorpha halys* were compared using multivariate methods provided by the PAST analysis package. Cluster analysis was based on the Bray–Curtis similarity index, nearest-neighbor. Cophenetic correlation coefficient was determined to assess the significance of obtained clusters.

## 3. Results

### 3.1. Male Genital Apparatus

**Figure 1:**
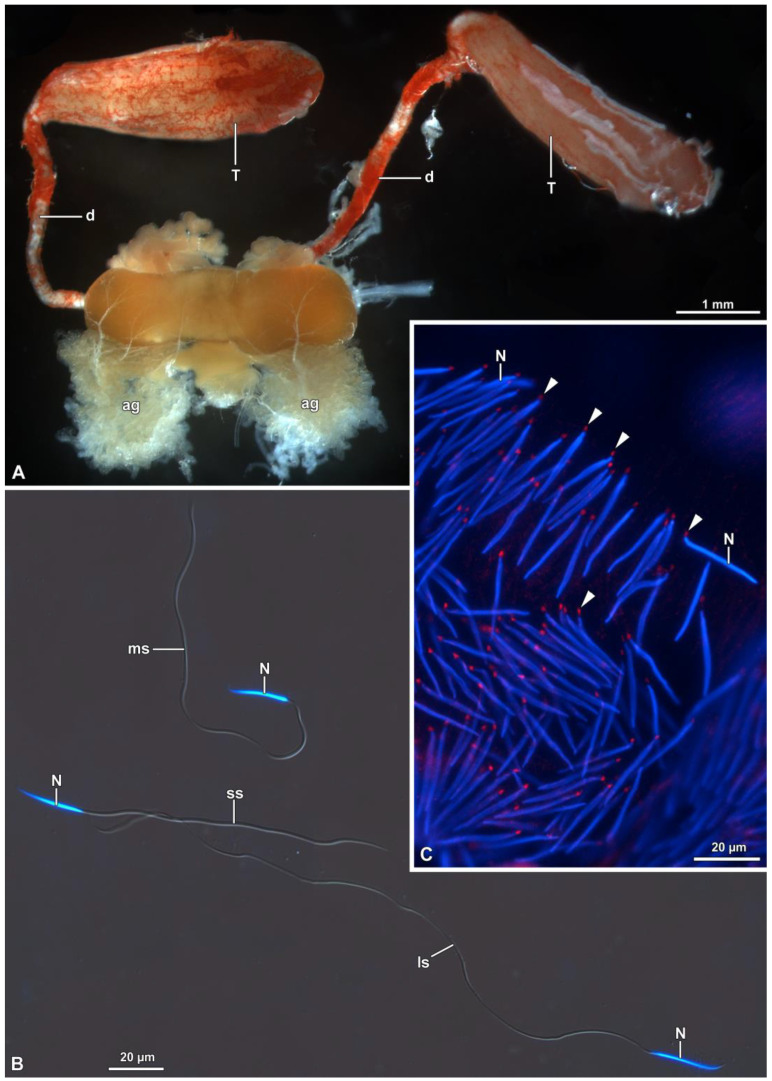
(**A**) General organization of the male genital apparatus of *Halyomorpha halys*. It is possible to observe the testes (T), the vasa deferentia (d) and the accessory glands (ag). (**B**) Mature sperm of different sizes: long sperm (ls), medium-size sperm (ms) and short sperm (ss). (**C**) Almost mature spermatids of *H. halys* observed with fluorescence microscopy after treatment with γ-tubulin. Note the nucleus (N) in blue, and fluorescent red dots corresponding to the centrioles at the posterior end of the nuclear material (arrowheads).

**Figure 2:**
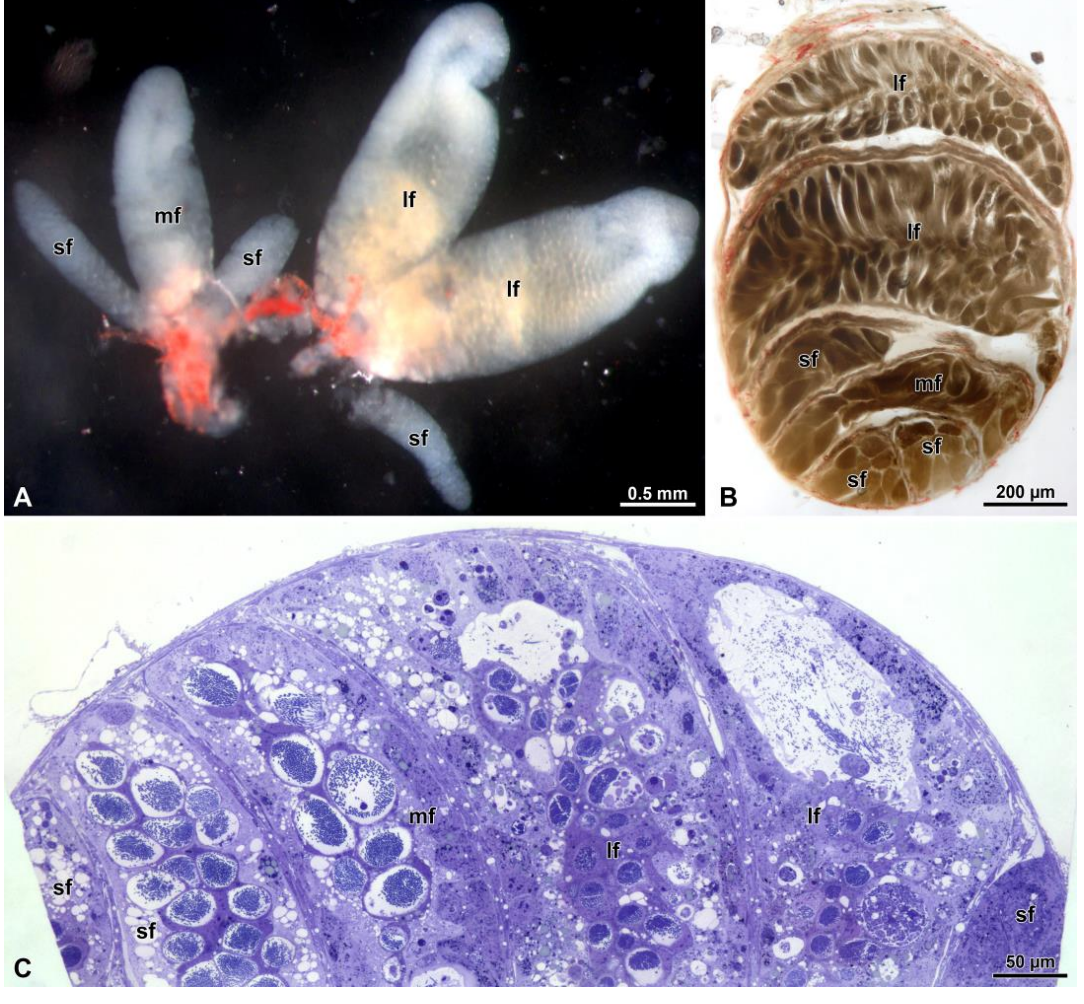
(**A**) Dissection of the testis showing the six different follicles. Two of them are larger, one is of medium size and three are smaller. (**B**) Section of the whole testis showing the six follicles of different sizes. (**C**) Semi-thin section of the testis showing the general arrangement of the sperm cysts. lf, large follicle; mf, medium follicle; sf, small follicle.

The male genital apparatus consists of two testes (dimensions: 3.5–3.7 mm × 0.7–1.1 mm), each made of 6 follicles, covered with a red-pigmented peritoneal sheath (Figure 1A and Figure 2A–C). The follicles vary in size: two are larger (about 13 mm × 4.5 mm), one is medium (9.6 mm × 3.3 mm) and two are smaller (about 6–9 mm × 1.2–2.2 mm). From each testis, a deferent vas, approximately 4–5 mm in length and 0.15–0.20 mm in width, originates (Figure 1A). Before fusing together to form the ejaculatory duct, the tubular section of the vasa deferentia enlarges to constitute the seminal vesicle and then receive the secretum of the accessory glands (Figure 1A).

### 3.2. Spermiogenesis

**Figure 3:**
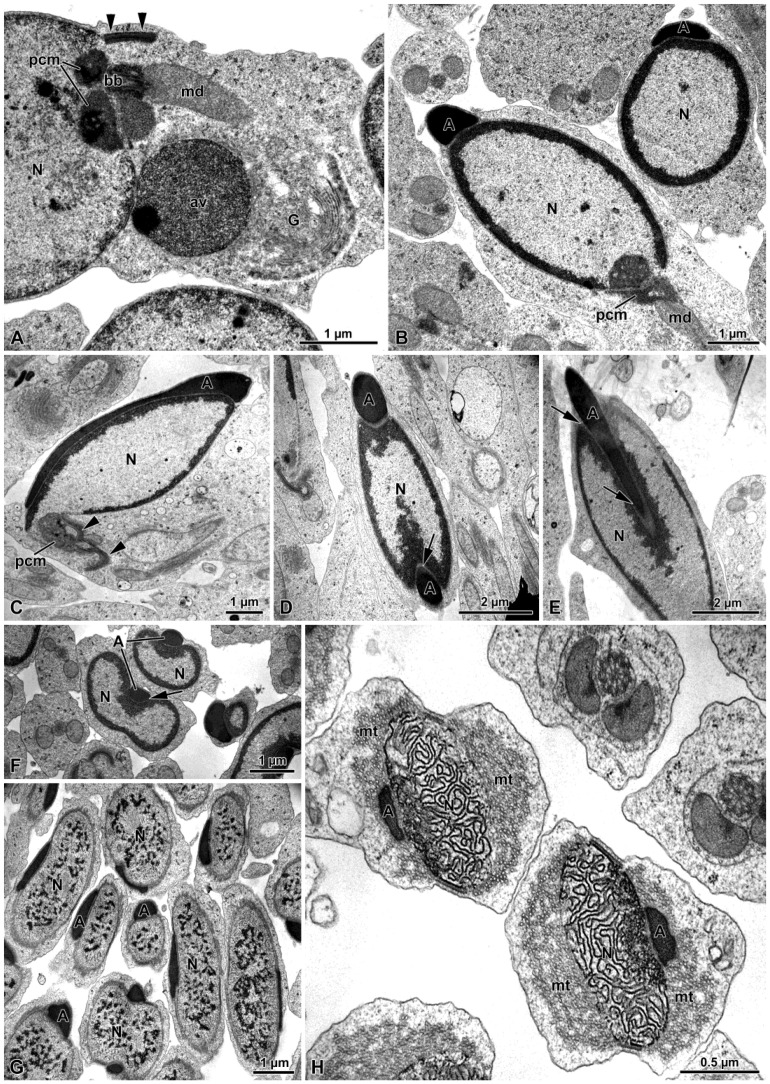
TEM sections of spermatids. (**A**) Transverse section of a young spermatid, in which it is possible to observe the Golgi apparatus (G), the acrosome vesicle (av), the nucleus (N), the mitochondrial derivative (md), the basal body (bb), the pericentriolar material (pcm) and part of the MTOC (arrowheads). (**B**–**F**) Sections of advanced spermatids showing the elongated acrosome (A) hosted in a longitudinal groove of the nucleus (arrows). N, nucleus; md, mitochondrial derivative; pcm, pericentriolar material; arrowheads, MTOC. (**G**,**H**): transverse sections of advanced spermatid with different chromatin condensation. Note in (**G**) a dotted condensation, while in (**H**) the chromatin assumes a filamentous appearance. N, nucleus; A, acrosome; mt, microtubules.

The acrosome vesicle originates from the Golgi apparatus in early spermatids (Figure 3A). At the beginning of its formation, such a vesicle has a circular shape of about 2 µm in diameter and a granular appearance. In this phase, this vesicle can be observed close to the mitochondrial derivatives and the pericentriolar material (PCM) (equivalent to what is also known as centriolar adjunct) (Figure 3A). In late spermatids, the acrosome consists of an electron dense material that extends from the apex to the basal part of the nucleus, reaching a length of about 5–7 µm (Figure 3B–H). At this stage, transverse sections of the nucleus show the presence of a groove running along the longitudinal part of the nucleus. This groove houses the acrosome (Figure 3D,F).

In early spermatids, the nucleus is approximately 5 µm long and has a globular shape, with no evidence of chromatin condensation observed (Figure 3A). As maturation proceeds, the chromatin condensates first, forming electron dense spots in a scattered manner (Figure 3G) and then it aggregates in irregular coiled filaments (Figure 3H). Transverse sections of the spermatid at this stage show that the nucleus and the acrosome are surrounded by numerous microtubules immersed in an opaque matrix (Figure 3H).

**Figure 4:**
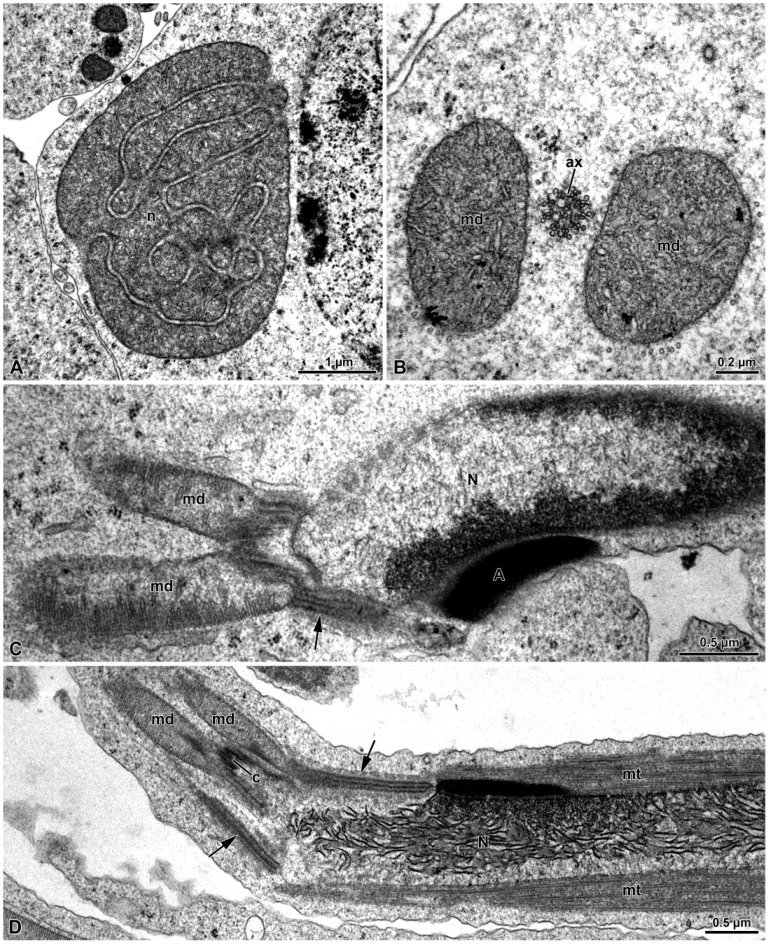
TEM section of spermatids. (**A**) The nebenkern (n). (**B**) The two mitochondrial derivatives (md) originated from the nebenkern; ax, axoneme. (**C**,**D**) Longitudinal section of the spermatid showing the acrosome (A), the nucleus (N), the two elongated mitochondrial derivatives (md) and the pericentriolar material organized as an early structured MTOC (arrows). Note the microtubules (mt) along the nucleus; c, centriole.

After the nebenkern formation, which occurs in early spermatids, the two mitochondrial derivatives are observed close to the basal body (Figure 4A–D), which, subsequently, give rise to the axoneme.

**Figure 5:**
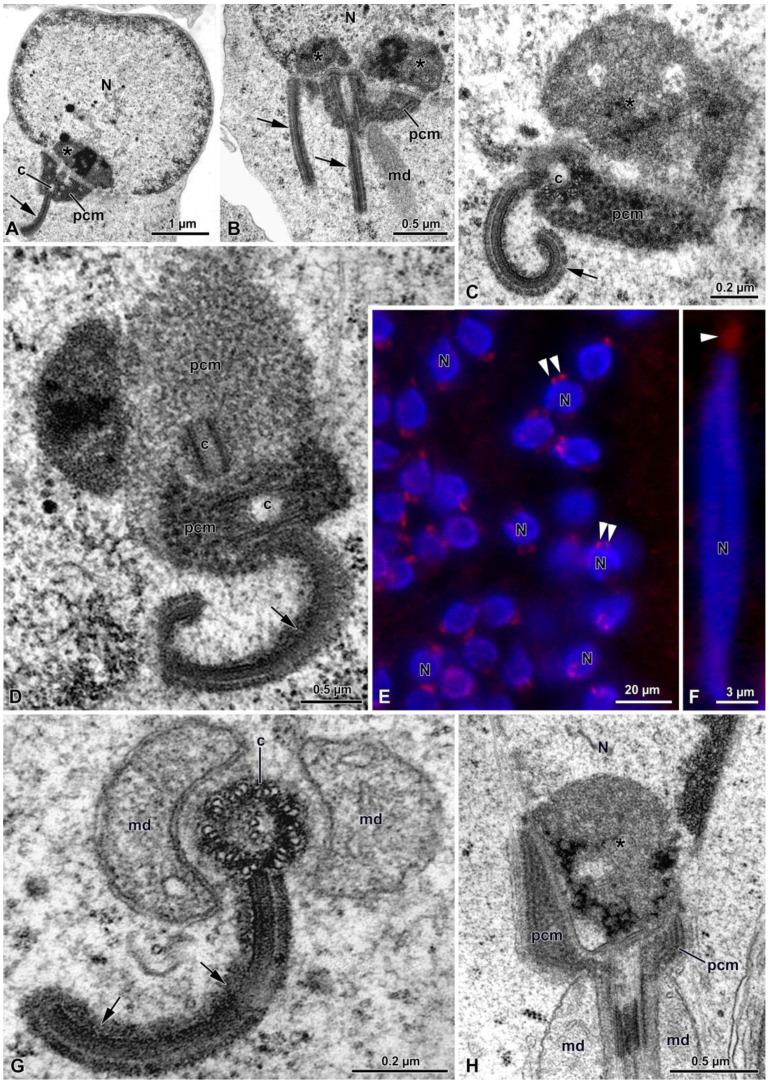
(**A**–**C**) Cross-section of the spermatids showing a roundish nucleus (N) with the centriole (c) immersed in the pericentriolar material (pcm). From the centriole originates an elongated, bent MTOC (arrows). Note the amount of electron-dense material filling the posterior end of the nucleus (asterisks); md: mitochondrial derivative. (**D**) Higher magnification of the centriolar region showing two centrioles (c) in orthogonal array immersed in two differently dense pericentriolar materials (pcm). The distal centriole gives rise to a bent MTOC (arrow). (**E**) Early spermatid after γ-tubulin and Hoechst staining. In red, the two structures containing γ-tubulin (centrioles and MTOCs; arrowheads) are visible close to the nucleus (N). (**F**) Almost-mature spermatids, in which only one centriole per spermatid is visible (arrowhead). N, nucleus. (**G**) Cross-section through the centriolar level showing the centriole (c) and the MTOC (arrows) together with the two mitochondrial derivatives (md). (**H**) Longitudinal section at the nuclear (N) posterior end in which the pericentriolar material (pcm) is visible. Note the amount of electron-dense material filling the posterior end of the nucleus (asterisk); md, mitochondrial derivatives.

Once the basal body is close to the nucleus, several processes occur: the basal body makes contact with the nucleus and the PCM, consisting of two different densities, and becomes evident around the organelle (Figure 5A–D). In one section of a spermatid, in the less electron-dense part of the PCM, another centriole of about 205 nm in length was visible (Figure 5D). Given its orthogonal position with respect to the distal centriole, it was considered to be the daughter, or proximal centriole (Figure 5D). The presence of both centrioles was also highlighted with immunofluorescence staining against γ-tubulin (5E). Afterwards, in advanced spermatids, such a proximal centriole is no longer visible in the process of maturation under TEM nor under immunofluorescence staining (Figure 1C and Figure 5F).

Within the nucleus, in correspondence to the basal body and the PCM, an amount of dense material leaning against the nuclear envelope is visible (Figure 5H).

**Figure 6:**
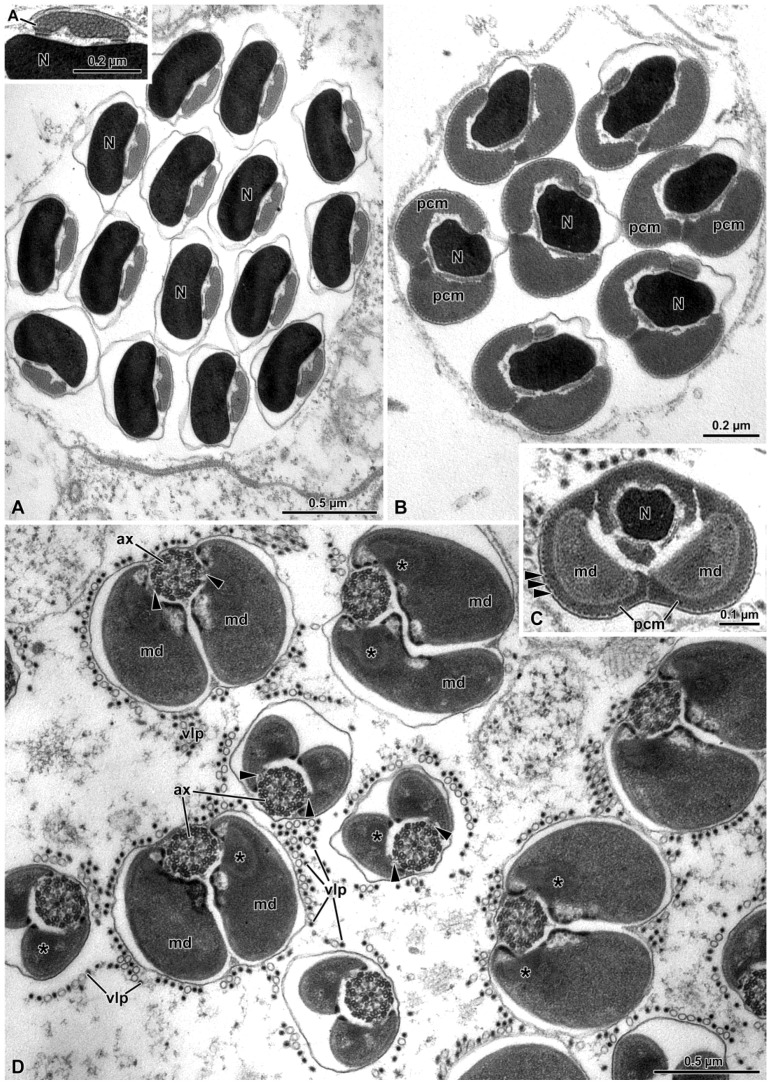
(**A**) Transverse sections of wild cyst reveal the characteristic arrangement of the crescent-shaped nucleus (N) with condensed chromatin and the slender acrosome (A) positioned lateral to the nucleus. The inset highlights the tubular material filling the acrosome. (**B**) Cross-section of the sperm cell at the posterior end of the nucleus, showing well-developed pericentriolar material (pcm) organized in two parts. N, nucleus. (**C**) Cross-section of the mature sperm at the nuclear posterior end level in which the two mitochondrial derivatives (md) are embedded within the pericentriolar material (pcm). Note the structured cortex of the pericentriolar material (arrowheads). N, nucleus. (**D**) Cross-section of the sperm flagella showing the axonemes (ax) connected by bridges (arrowheads) to the mitochondrial derivatives (md). The mitochondrial matrix towards the axoneme is crystallized (asterisks). Note the variation in sizes of the mitochondrial derivatives; vlp: virus-like particles.

The material surrounding the centriole and the apical part of the mitochondrial derivatives allows the young axoneme to tighten with the nucleus. In the mature sperm, a layer of the PCM envelopes the nuclear envelope that is thinning from the basal to the apical part (Figure 5H and Figure 6B,C).

In some sections of spermatids, a lamellar plaque approximately 750 nm in length was observed originating from the external side of the centriole triplets (Figure 5A–D,G). This plaque, whose depth was not assessed, is assumed to be a microtubule organizing center (MTOC). It consisted of four overlapping layers with varying electron densities and typically exhibited a bent form(Figure 5A–D,G). During maturation, the bending plaque appeared to contact the nuclear envelope, forming a loop (Figure 5B–D,G). However, the origin of this process was unclear. In mature sperm, the MTOC was no longer visible.

When mature, the nucleus exhibits a slightly crescent shape in the apical part (Figure 6A) and a cylindrical shape in its basal part, where it is surrounded by the PCM (Figure 6B,C). At lower level, the PCM encircles both the nucleus and the mitochondrial derivatives (Figure 6C). The length of mature nuclei ranges from about 21 µm to 29 µm (Figure 1B and Figure 6A).

In mature spermatozoa, acrosomes measure between 2.3 and 6.5 µm in length and consist of tightly packed tubules, visible in transverse sections (Figure 6A, inset).

The tail exhibits the highest variability in size among sperm component, ranging from about 110 µm to 260 µm in length. Here, the flagellar axoneme, characterized by the classical 9 + 9 + 2 microtubular array, features two bridges connecting the mitochondrial derivatives to the intertubular material (Figure 6D).

**Figure 7:**
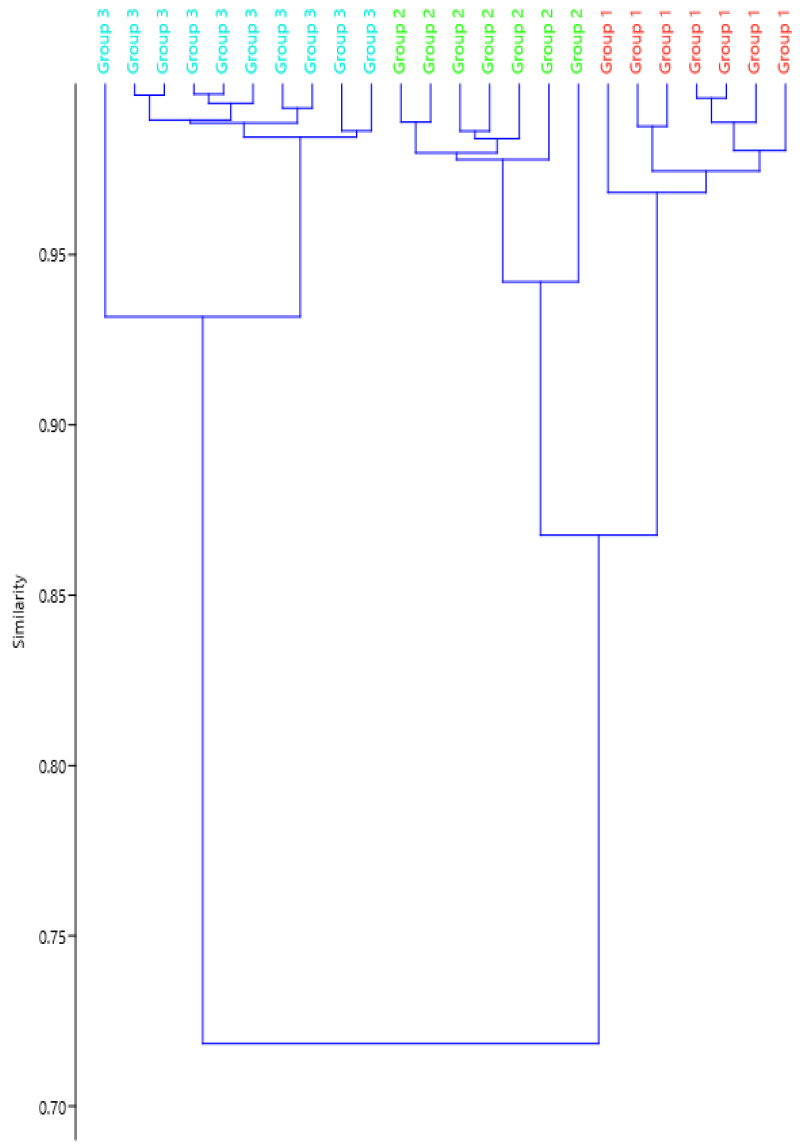
Cluster analysis of the sperm length in *Halyomorpha halys* showing the degree of similarity of the different length categories.

Based on the measures of 36 mature spermatozoa, we identified three length categories: 142.83 ± 0.94 SE; 177.94 ± 2.92 SE and 277.90 ± 2.92 SE. The lengths of these three categories (small, medium and large) as well as the differences in length among the acrosome, nucleus and tail are reported in Table 1 (Cophen. corr. 0.9383) and in Figure 7.

The transverse sections of the mature sperm reveal occasional variations in diameter, with the mitochondrial derivatives sometimes being twice as large as the usual sperm diameter (Figure 6D).

### 3.3. Γ-Irradiation

**Figure 8:**
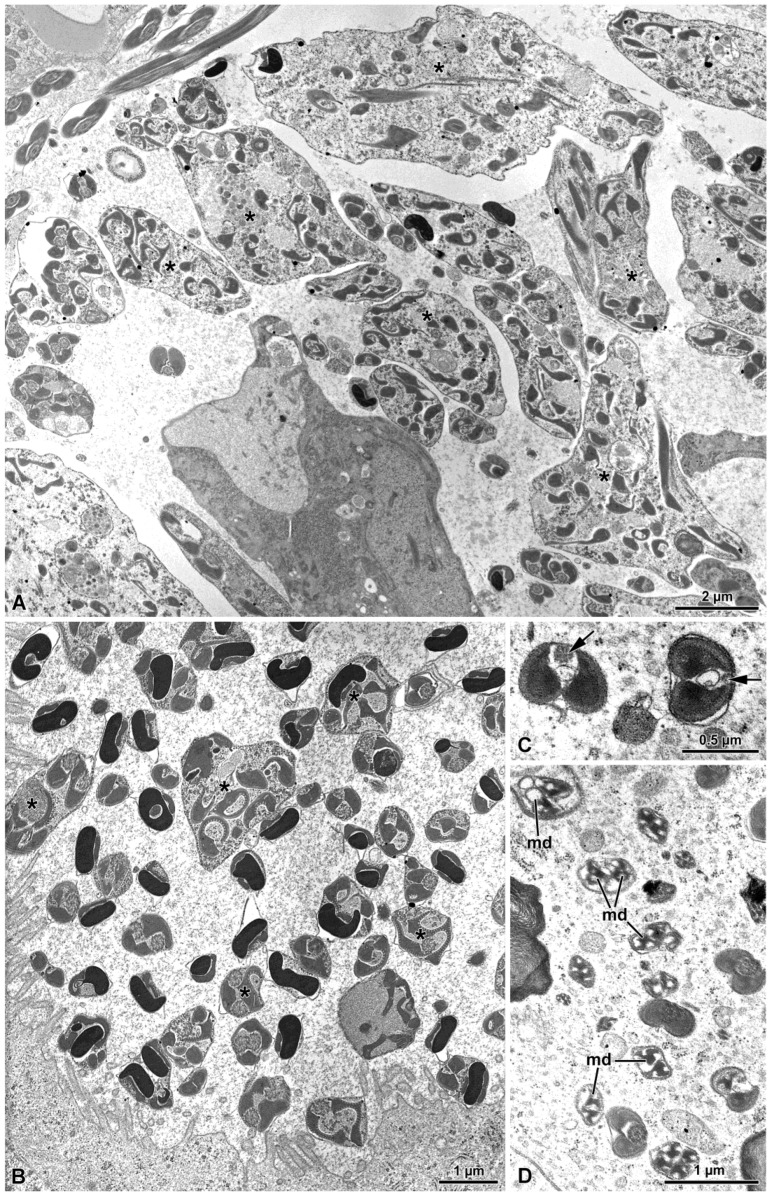
Cross-section through irradiated testis. (**A**) Testis showing several cysts with disorganized germ cells (asterisks). (**B**–**D**) Spermatids at different maturation stages with different types of degeneration (e.g., incomplete cytodieresis (asterisks)) with alterations of different sperm components, lack of axonemes (in (**C**) (arrows) and in (**D**) disrupted mitochondrial derivatives (md).

The observations of sperm from newly emerged adults, γ-irradiated at 40 Gy, revealed, only in some cysts, some morphological differences in the organelle maturation and sperm arrangement within the cyst cell (Figure 8A–D). Notably, disarray in sperm organization was observed, including incomplete cytokinesis resulting in organelle duplication within individual sperm cells. Additionally, incomplete reabsorption of cytoplasmic material was noted in some instances, along with the absence or degeneration of axonemes and/or mitochondrial derivatives (Figure 8A–D).

### 3.4. Virus-Like Particles

**Figure 9:**
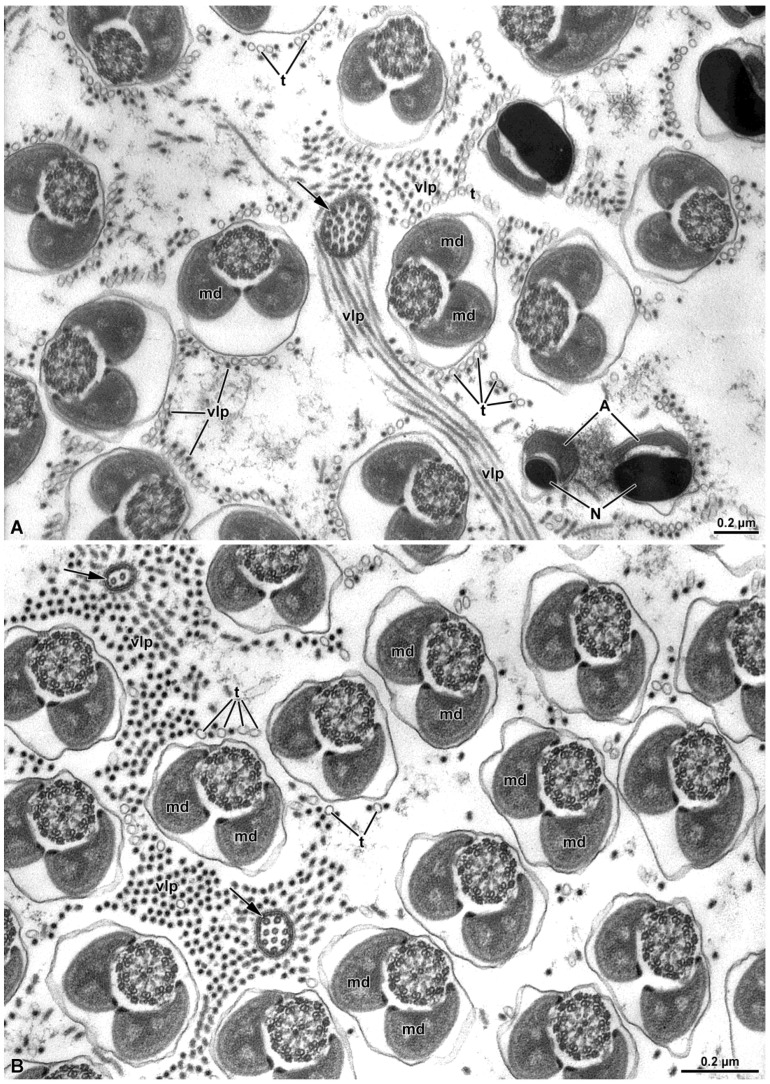
(**A**,**B**) Cross-section of the sperm showing uniform dimensions of the mitochondrial derivatives (md). In (**A**), various levels of the sperm are depicted: in the lower right corner of the figure, note the most anterior region of the nucleus (N) and acrosome (A). At the tail end (arrows), disorganization of the axonemal microtubules is evident. Among the sperm cells, numerous virus-like particles (vlp) are visibly free in the intercellular space. The virus-like particles exhibit a filamentous appearance and are filled with electron-dense material. Also, tubules (t) are present and free in the intercellular space.

During the observation of the sperm in both treated and untreated adult males of *Halyomorpha halys*, we noted the presence of rod-shaped virus or virus-like particles freely distributed in the extracellular space among the sperm cells and other tubules (Figure 9A,B). The virus-like particles exhibited a tubular appearance, and in transverse sections displayed dense material within them. While these structures were commonly observed in males, they were never detected in sperm cells obtained from the female spermathecae.

## 4. Discussion

The investigation of the sperm morphology in *Halyomorpha halys* revealed characteristic variations in sperm length. Such a polymorphism is known to occur in Pentatomidae and has already been observed in the subfamily Pentatominae [22] to which *H. halys* belongs. Following the categorization performed [22], we reported the existence of three categories of sperm length in the Results section of the present contribution. However, our data do not conclusively support the grouping into three categories, as reported in that paper. In fact, the most evident finding is a clear separation in length between longer sperm cells and the combined medium-sized + smaller ones, with a diversity of about 29%; the separation between medium-sized and smaller ones has a degree of diversity limited to 14%, thus indicating that they are quite similar to each other. Therefore, a more comprehensive interpretation of the cluster analysis suggests the existence of two main categories of length instead of three: the first group contains sperm with lengths ranging from 130 to 190 µm and the second group contains sperm with lengths ranging from 250 to 290 µm. In addition, we observed that the variation in sperm length is primarily due to differences in the length of the tail, while the length of the head remains steadier. Furthermore, in cross-sections, we also observed a variation in the diameter of the sperm, with the mitochondrial derivatives being twice as large as those of sperm with a common size. Whether this length variation results from aberrant sperm maturation warrants further investigation.

Our study showed that each testis contained six follicles of different dimensions: two larger, one medium-sized and three narrow follicles. However, we could not confirm that the sperm cells of different lengths originated from different follicles, nor that only the longer sperm were responsible for fertilization [22].

Although the spermiogenesis in Heteroptera has been studied in several species (e.g *Pyrrochoris apterus* [19]; *Eurygaster intergriceps* [20]) and the sperm features of the Pentatomidae have been investigated in various studies [23,25], the ultrastructural exploration of *H. halys* spermiogenesis deserves particular attention. This is due to two reasons: firstly, *H. halys* is a significant pest, and secondly, understanding the ultrastructural changes induced by the SIT is essential for its effective implementation.

SIT is a minimally toxic pest control method in which adult males are irradiated to produce sperm cells that cannot fertilize oocytes. This technique has two main requirements: (1) the irradiated males must remain alive and sexually competitive with wild males for at least two weeks after irradiation and (2) the sperm must remain sterile for the male’s entire lifespan after irradiation [26]. Moreover, if last-male sperm precedence occurs among sexual behaviors, the effectiveness of this control technique is enhanced. With regard to the latter statement, however, it should be taken into account that even in species in which last-sperm precedence is known to occur (e.g., *Bagrada hilaris*—Heteroptera: Pentatomidae), a sperm mixing from different males is sometimes possible at post-copulatory level [27].

In previous experiments, the irradiation of 40 Gy was tested as a candidate dose for applying SIT to *Halyomorpha halys* since treated individuals achieved a high sterility level of more than 95%. Treated individuals were not affected in sexual behavior and had a life span of about 2–3 weeks after treatment [28]. This is why the observations of the treated insects in the present work have investigated this level of irradiation.

The ultrastructure of sperm cell maturation shows the characteristics typical of the Heteroptera group along with some novelties. Among the features already known, we can list the presence of an atypical MTOC with a laminar shape, similar to what was observed in *Pyrrochoris apterus* [19], *Acrosternum aseadum* and *Euschistus heros* [25], *Coptosoma scutellatum* [21] and *Eurygaster intergriceps* [20]; the presence of a bridge between the mitochondrial derivative and the axonemes; the absence of accessory bodies and the presence of two or three crystallized bodies within the mitochondrial derivatives [18].

The acrosome of *H. halys* exhibits a unique morphology. It extends along the length of the nucleus, originating at its basal region and reaching the apex. In some longitudinal sections, a groove along the nucleus is visible, potentially facilitating acrosomal adhesion. Upon maturation, the acrosome becomes filled with tightly packed tubules.

Transmission electron microscopy (TEM) unveiled a unique feature in *H. halys* spermatids: the presence of two orthogonal centrioles, each embedded within a distinct electron-dense PCM. The presence of two differently electron-dense PCMs was also observed in *Coptosoma scutellatum*, although the presence of a second centriole was not spotted [21]. The investigation with a fluorescent anti γ-tubulin marker reported the presence of two spots. However, it is worth questioning what these γ-tubulin spots indicate. It is possible that they mark the two centrioles. Nevertheless, sometimes one spot is apparently slightly longer than the other. This could mean that the longer spot of γ-tubulin represents the distal centriole and the atypical MTOC with a bent-plaque shape, while the shorter one reveals the temporary proximal centriole. Subsequently, in mature spermatids, only one spot is visible with both immunolabeling and TEM.

This fact deserves a discussion: in most animals, spermatids have two centrioles (the distal and proximal one), but insect spermatids were thought to contain only one centriole [29,30,31], which functionally works as a distal centriole, giving rise to the axoneme.

In early spermatids of insects, the centrosome is typically composed of two distinct elements: a single centriole and a cloud of electron-dense pericentriolar material (PCM) surrounding it. During spermiogenesis, the centriole undergoes structural modifications to give origin to the flagellar axoneme. At the same time, γ-tubulin, which is a component of the PCM, plays a crucial role in microtubule nucleation, which is essential for the elongation and shaping of various spermatid structures [32]. The latter feature was also observed in *H. halys*, where PCM gave origin to a thick layer of microtubules that encased the nucleus to constitute the manchette needed for the nuclear shaping.

In *Drosophila melanogaster*, a centriole was identified close to the distal centriole that was reminiscent of the proximal centriole (PCL) [33]. It could be suggested that also in our study the fluorescent marker for γ-tubulin revealed, at a certain stage of maturation, the material of both the PCL and the distal centriole together with the MTOC. Afterwards, however, the PCL was reabsorbed, and its presence was no longer detectable by immunofluorescence.

The description of atypical centrioles and MTOCs in insects occurred several times [34,35,36]; however, the first description of such an MTOC in spermatids was performed by Godula (1979) [19], who described this type of cytoplasmic component in the differentiating firebug spermatids. Such an MTOC usually exhibits the shape of a bent ribbon consisting of four layers of different electrondensity. As expected, this MTOC contains γ-tubulin from which, apparently, microtubules are enucleated. This structure might be considered as a structured extension of the PCM.

Beyond its role in microtubule enucleation, this MTOC might also have a mechanical function. It could potentially facilitate contact between the basal body and the nucleus, and/or tether mitochondrial derivatives to the axoneme, thereby establishing the characteristic axoneme–mitochondrial derivative linkage observed in Heteroptera.

Cross-sections of mature sperm reveal a conspicuous mass of PCM enveloping the lower part of the nucleus and upper part of the mitochondrial derivatives. This discrete mass likely functions to connect the sperm from head to the tail.

Irradiated adult specimens revealed a lack of uniform cytological effects in the cysts. This variation likely stems from the cell division phase at the time of treatment. Since irradiation targets DNA integrity, the most pronounced morphological damage is expected when cells are affected during a specific stage of division. Therefore, the observed diversity in damage levels can be attributed to the slight asynchrony in sperm cell maturation within different germ cysts.

Irradiated males exhibit a range of sperm abnormalities, including disorganized cyst structure, incomplete cell division leading to multi-organelle copies, failure of cytoplasmic reabsorption, and lack of axonemes or mitochondrial derivatives. These defects contribute to render the sperm incapable of fertilization.

However, it is important to note that sometimes processes of apoptosis and/or cyst cell disorganization may naturally occur in wild individuals as well [37]. Therefore, we cannot exclude that, at least in some limited cases, the observed disorganization of the cyst cells is the result of the natural degeneration that sometimes happens during the spermatogenic process.

Cross-sections of vasa deferentia showed mature sperm cells intermingled with hundreds of rod-like structures and empty tubules free in the extracellular space. The former structures, filled with an electron-dense material, are reminiscent of viruses or virus-like material. In a recent paper on the virome of *H. halys* [38], several viruses were identified. Furthermore, similar structures, with an elongated shape were observed in *Raphigaster nebulosa* (Heteroptera: Pentatomidae) and in *Pyrrochoris apterus* (Heteroptera: Pyrrochoridae) [24,39]. In both cases, those particles were considered as rhabdoviruses, despite their different lengths. The function of these viral materials is unknown: their absence from the spermathecae of mated females suggests that they do not play a role in fertilization. Furthermore, these structures were observed in both irradiated and non-irradiated sperm cells.

In conclusion, this study highlighted the ultrastructural characteristics of the spermiogenesis in *H. halys*, reporting novel findings on the centriolar region of the spermatids and showing the impact that 40 Gy irradiation has on the maturation of sperm ultrastructure.

## Figures and Tables

**Table 1:** Lengths of the different components of mature sperm cells in *Halyomorpha halys* (n = 36). SE = standard error.

		Length	SE
Acrosome	Small	4.53	0.35
	Medium	3.54	0.39
	Large	3.48	0.30
Nucleus	Small	25.59	0.68
	Medium	22.69	0.66
	Large	25.83	0.47
Flagellum	Small	113.71	0.74
	Medium	151.71	2.87
	Large	249.32	2.85
Total length	Small	142.83	0.94
	Medium	177.94	2.92
	Large	277.90	2.92

## Data Availability

Data are available upon request.
